# P-1883. Online Medical Education Effectively Improves Clinician Knowledge, Competence and Confidence in Implementing Antimicrobial Stewardship Practices When Diagnosing and Managing Suspected Bloodstream Infections with Available Molecular Rapid Diagnostic Tests

**DOI:** 10.1093/ofid/ofaf695.2052

**Published:** 2026-01-11

**Authors:** Arun S Nair, James Martorano, Roderick Smith

**Affiliations:** Medscape Education, TARRYTOWN, New York; Medscape EDucation, New York, NY; Medscape, New York, New York

## Abstract

**Background:**

This study evaluated the impact of online CME on knowledge, competence, and confidence in integrating molecular rapid diagnostic tests (mRDTs) and antimicrobial stewardship (AMS) strategies for diagnosing and managing suspected bloodstream infections (BSIs).

**Methods:**

We developed an on-demand 30-minute online CME activity presented by 3 experts with accompanying slides. Educational impact assessed for learners completing pre-/post-assessment questions using a matched pre-/post-assessment design. A paired samples t-test measured significance in overall correct responses and confidence ratings, while a McNemar test assessed question-level changes (*P* < .05). Confidence was rated on a 5-point Likert scale. Data were collected from 01/2025 - 03/2025.

**Results:**

As of 03/2025, the curriculum reached approximately 5,800 learners, mainly Infectious Disease (ID) & Critical Care (CC) Specialists. Pre-/post-assessment data indicated significant learning improvements (P < .001), with a 35% and 42% increase in knowledge & competence related to integrating rapid testing for optimizing BSI treatment among ID (n=105) and CC (n=52) Specialists, respectively. Additionally, both groups experienced about a 28% boost in confidence regarding their ability to optimize management of suspected BSIs.

Additional learner insights include:

• Despite modest learning improvements, the education reinforced core values for integrating rapid blood panels in over 50% of ID and CC specialist groups

• > 20% of learners still show gaps in antibiotic stewardship, continuing empiric treatments or switching to ineffective therapies despite appropriate test results

• ∼40% of learners found the subject matter new, highlighting foundational gaps clinicians have with the availability and clinical utility of mRDTs and novel AMS testing strategies

**Conclusion:**

These findings highlight the importance of online CME/CE in promoting AMS strategies for suspected BSIs using available mRDTs. Future educational initiatives should deepen learners' understanding of BSI panel characteristics and optimizing treatment decisions through effective stewardship, while also raising awareness of the clinical utility of BSI panels and antimicrobial susceptibility testing via case-based activities.

**Disclosures:**

All Authors: No reported disclosures

